# Population genetic structure of zoonotic *Toxoplasma gondii* in China revealed using multilocus sequence typing

**DOI:** 10.1016/j.soh.2026.100162

**Published:** 2026-05-27

**Authors:** Yi-Tian Fu, Yu-Na Xiao, Xi-Long Yi, Hany M. Elsheikha, Bei-Bei Zhou, Meng-Ling Deng, Feng-Cai Zou, Xing-Quan Zhu, Guo-Hua Liu

**Affiliations:** a Research Center for Parasites & Vectors, College of Veterinary Medicine, Hunan Agricultural University, Changsha 410128, Hunan, China; b Faculty of Medicine and Health Sciences, School of Veterinary Medicine and Science, University of Nottingham, Sutton Bonington Campus, Loughborough LE12 5RD, UK; c The Yunnan Key Laboratory of Veterinary Etiological Biology, College of Veterinary Medicine, Yunnan Agricultural University, Kunming 650201, Yunnan, China; d Shanxi Key Laboratory of Animal Disease Research, Prevention and Control, College of Veterinary Medicine, Shanxi Agricultural University, Taigu 030801, Shanxi, China

**Keywords:** *Toxoplasma gondii*, Population structure, Cryptic diversity, Rare lineage

## Abstract

**Background:**

The zoonotic pathogen *Toxoplasma gondii* exhibits a diverse global population structure, with a few dominant lineages primarily in the Northern Hemisphere. However, reliance on low-resolution, restriction-based genotyping methods has created a “resolution ceiling,” potentially masking hidden genetic diversity and complex transmission dynamics.

**Methods:**

In this study, we combined conventional PCR-restriction fragment length polymorphism (RFLP) screening with high-resolution Sanger sequencing targeting 16 genetic markers to unravel the fine-scale epidemiology of 96 *T. gondii* DNA samples collected from various hosts (including pigs, cats, sheep, birds, bats, and captive wildlife) across 12 provinces and regions in China.

**Results:**

Our analysis identifies ToxoDB#9 as the dominant lineage (41/96 samples), revealing substantial intra-clonal diversity within this lineage. We report the North American sylvatic ToxoDB#5 (Haplogroup 12) lineage in a captive caracal in China, documenting the presence of this rare lineage outside its previously recognized range, although its public health significance remains uncertain. Population genetic analyses show high haplotype diversity consistent with clonal diversification with limited geographic structuring.

**Conclusion:**

Our study provides an updated baseline for *T. gondii* genetic diversity in China and supports the value of systematic molecular monitoring within a One Health framework, with whole-genome sequencing required to confirm introduction scenarios, resolve transmission routes, and assess recombination.

## Graphical abstract

High-resolution 16-locus multilocus sequence typing (MLST) of 96 *Toxoplasma gondii* DNA samples from multiple hosts across China revealed dominance of the ToxoDB#9 lineage with cryptic intra-lineage diversity, high haplotype diversity but limited geographic structuring, and detection of the rare ToxoDB#5 lineage in captive wildlife. These findings support systematic One Health molecular monitoring and future whole-genome sequencing to resolve isolate origins, transmission routes, and recombination.

**Figure:**
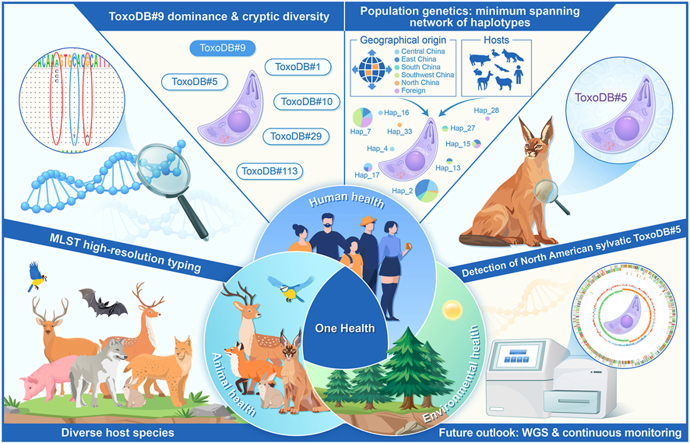

## 1 Introduction

The global epidemiology of *Toxoplasma gondii*, a ubiquitous zoonotic protozoan, reveals substantial genetic diversity dominated by a few key lineages across the Northern Hemisphere [[1], [2], [3]]. ToxoDB#1 and #3 (collectively known as Type Ⅱ) and #2 (Type Ⅲ) prevail in Europe, many regions of Africa, and North America, while the ToxoDB#9 (Chinese 1) lineage is dominant in East Asia [[4], [5], [6]]. However, these phylogeographic patterns indicate relative predominance rather than strict geographic isolation, with occasional detections outside presumed core regions increasingly reported. In the Anthropocene era, intensified animal movements, livestock trade, and human-mediated translocations may increase opportunities for long-distance dispersal and the geographic mixing of lineages, thereby blurring traditional regional genetic signatures [7,8].

Despite this dynamic landscape, understanding fine-scale population processes remains constrained by a critical bottleneck: the widespread reliance on PCR-restriction fragment length polymorphism (RFLP) genotyping [9,10]. While robust for broad lineage classification, this method interrogates only a limited number of restriction sites, imposing a “resolution ceiling” that masks intra-genotypic diversity and obscures the subtle evolutionary dynamics driven by recent anthropogenic mixing [11]. This limitation hampers our ability to fully capture the complex population structure of *T. gondii* and track emerging strains.

Moreover, the prevailing genotyping standards have contributed to a genotype–phenotype paradox in *T. gondii*. Even among isolates assigned to broadly similar genotypes, marked differences in virulence can occur, suggesting that conventional PCR-RFLP methods fail to capture biologically relevant variation, particularly in polymorphic virulence-associated effectors [[12], [13], [14]]. Recent genomic studies have begun to address the gap in whole genome sequencing (WGS) data for Asian isolates [15,16]. Notably, Ihara et al. [16] revealed multiple ancestral links between Far East Asian and American *T. gondii* strains, demonstrating that haploblocks from Chinese lineages (e.g., Haplotype 13) are introgressed within North American lineages. These findings highlight shared ancestry and historical connectivity among *T. gondii* populations across continents as revealed by genome-wide haploblock patterns. However, the scarcity of routine high-resolution surveillance data remains a challenge, leaving a critical spot in our understanding of whether and how rare lineages and genetic strains of uncertain phenotypic relevance are maintained within local livestock populations.

To address these challenges and overcome the limitations of traditional genotyping, we employed a high-resolution 16-locus multilocus sequence typing (MLST) approach on *T. gondii* isolates collected from diverse hosts across 12 provinces and regions in China. This strategy transcends the constraints of restriction-site-based methods, allowing us to reveal cryptic polymorphisms within the dominant ToxoDB#9 lineage.

## 2 Methods

### 2.1 Geographical origins of *T. gondii* strains

The 91 *T. gondii*-positive samples originated from 12 provinces and regions in China, including Beijing, Gansu Province, Jilin Province, Henan Province, Hubei Province, Jiangxi Province, Anhui Province, Fujian Province, Jiangsu Province, Guangdong Province, Guangxi Zhuangzu Zizhiqu (also known as Guangxi Zhuang Autonomous Region), and Yunnan Province. These sampling sites covered North, Central, East, South, and Southwest China. Detailed information on the geographical origin, host species, and genotype of each isolate is provided in Table S1. Because the samples were collected opportunistically, these strains do not represent a systematic geographical survey of *T. gondii* in China.

### 2.2 Sample collection and DNA extraction

A total of 96 *T. gondii*-positive samples (including five reference isolates that yielded complete sequence data), previously confirmed by semi-nested PCR targeting the 131 bp *B1* gene [17,18], were included in this study. These samples were obtained from a variety of animal hosts, including pigs, cats, sheep, birds, bats, and captive wildlife (Table S1). Samples were obtained through opportunistic sampling during routine veterinary inspections and clinical diagnoses. The sample sizes for certain species (e.g., bats, captive wildlife) were limited due to the scarcity of positive materials, the low prevalence of *T. gondii* infection in these hosts, and logistical/ethical constraints. Therefore, host-specific prevalence was not inferred from this dataset. Genomic DNA was extracted using the QIAamp DNA Micro Kit (Cat No. 56304; QIAGEN, Hilden, Germany) according to the manufacturer's protocol. To ensure accuracy and reproducibility, DNA from eight well-characterized reference strains (RH, PTG, CTG, MAS, TgCgCa1, TgCatBr5, TgCatBr64, and TgRsCr1) was included as positive controls for genotyping and sequencing, while DNA-free water served as the negative control.

### 2.3 Multilocus amplification and sequencing

Genotyping targeted 16 genetic loci, comprising 12 standard PCR-RFLP markers (surface antigen 1 [*SAG1*], 5′-*SAG2*, 3′-*SAG2*, alter. *SAG2*, *SAG3*, beta-tubulin [*BTUB*], dense granule antigen 6 [*GRA6*], C22–8, C29–2, L358, protein kinase 1 [*PK1*], and *Apico*), two housekeeping genes (hypoxanthine-xanthine-guanine phosphoribosyltransferase [*HP*, also known as HPRT/HXGPRT] and elongation factor [*EF*]) introns, and two apicoplast genes (*Apico1* and *Apico2*) (Table S2). To maximize sensitivity, a multiplex nested PCR approach was employed for the 12 PCR-RFLP markers: the first round consisted of two multiplex reactions each amplifying six loci, followed by individual nested PCRs for each marker. The housekeeping and apicoplast loci were amplified separately using standard nested or semi-nested PCR protocols. All PCR products were purified and subjected to bidirectional Sanger sequencing. Sequence assembly was performed using Chromas Pro (version 2.1), and alignments were conducted with MEGA (version 11.0) [19].

### 2.4 *In silico* genotyping and single nucleotide polymorphism (SNP) analysis

To facilitate comparison with conventional genotyping methods, *in silico* RFLP analysis was conducted by virtually digesting the sequenced amplicons using standard restriction enzymes within SnapGene (https://www.snapgene.cn/). The resulting virtual banding patterns were then compared to those of reference strains to assign genotypes (e.g., ToxoDB#9). Furthermore, full-length sequences were aligned to identify SNPs beyond restriction sites, enabling assessment of the enhanced resolution provided by sequence-based analysis relative to traditional virtual RFLP.

### 2.5 Population genetic and phylogenetic analyses

Sequences from all 16 loci were concatenated for each isolate, resulting in a dataset of approximately 7062 base pairs per sample. Genetic diversity indices, including haplotype diversity (Hd) and nucleotide diversity (π), were calculated using DnaSP (version 6) [20]. To visualize haplotype relationships, a minimum spanning network was generated using PopART (version 1.7) [21]. Discriminant analysis of principal components (DAPC) was used to identify genetic clusters without assuming Hardy–Weinberg equilibrium, making it suitable for predominantly clonal organisms such as *T. gondii*. Genetic data were initially transformed using principal component analysis (PCA), and clusters were subsequently identified using discriminant analysis (DA). The optimal number of clusters (*K*) was determined using the Bayesian Information Criterion (BIC), and the number of retained principal components was selected based on cross-validation to avoid overfitting. DAPC was performed using the “adegenet” package implemented in R (version 3.4.0) [22]. To investigate fine-scale population structure and patterns of strain migration within a single lineage, Analysis of Molecular Variance (AMOVA) was performed exclusively on isolates belonging to the ToxoDB#9 genotype (*n* = 41). Samples were grouped according to their geographical origins. Genetic differentiation was assessed using GenAlEx (version 6.51) [23] to partition variance components among and within populations. Finally, phylogenetic trees were constructed using Maximum Likelihood (IQ-TREE version2) [24] based on best-fit substitution models selected by PartitionFinder (version 2) [25].

## 3 Results

### 3.1 Dominance of ToxoDB#9 and detection of North American lineages

**Fig. 1:**
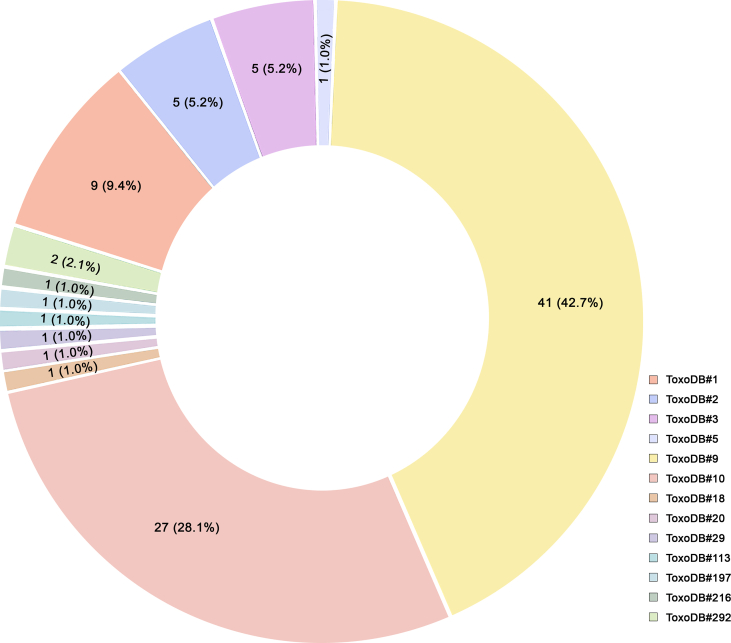
Distribution of *Toxoplasma gondii* genotypes identified in this study, showing the frequency of dominant and rare lineages across sampled provinces and regions in China.

*In silico* RFLP analysis of the 12 canonical markers successfully genotyped all 96 DNA samples, identifying 13 distinct genotypes (Fig. 1; Table S1). The ToxoDB#9 (Chinese 1) lineage was overwhelmingly dominant, representing 42.7% of isolates (*n* = 41) (Fig. 1) and displaying widespread distribution across all sampled provinces and major hosts, including pigs, voles, and domestic cats. These results confirm ToxoDB#9 as the predominant lineage in our sampling, consistent with prior reports from Chinese mainland. Importantly, high-resolution screening revealed the presence of the ToxoDB#5 (HG12) genotype in a captive caracal (*Caracal caracal*) from Henan province (Table S1). Additionally, analysis of stray cats from Guangzhou revealed marked genetic heterogeneity, with isolates corresponding to unique genotypes (ToxoDB#18, ToxoDB#29, and ToxoDB#113). In contrast, captive macropods were primarily infected with ToxoDB#292, with a single isolate identified as ToxoDB#3 (Table S1).

### 3.2 Cryptic intra-genotypic polymorphisms unmasked by MLST

While *in silico* RFLP analysis produced profiles fully consistent with conventional methods, direct nucleotide sequencing revealed substantial “hidden” diversity. To characterize the molecular signature of the dominant Chinese lineage, sequences from ToxoDB#9 isolates were aligned against the archetypal Type Ⅱ reference strain ME49 (Table 1). Despite identical RFLP patterns, ToxoDB#9 isolates exhibited fixed SNPs at multiple non-restriction sites, highlighting divergence from the global archetype. Specifically, stable mutations at loci L358, *BTUB*, and *PK1* distinguished the ToxoDB#9 lineage from ME49, confirming its status as a distinct genotype closely related to Type Ⅱ lineages.

**Table 1:** Characterization of cryptic SNPs in ToxoDB#9 vs Type Ⅱ (ME49).

Locus	RFLP status (Type Ⅱ)	Nature of polymorphism^a^	Number of SNPs	Molecular signature (base change)^b^	Biological implication
L358	Identical	Non-restriction site	1	170 bp (A→G)	Distinguishes ToxoDB#9 from other Type II strains
*PK1*	Identical	Non-restriction site	4	59 bp (G→A) 150 bp (C→T) 657 bp (G→A) 767 bp (C→G)	High resolution marker for Chinese Type II variants
C22-8	Identical	Non-restriction site	4	8 bp (G→C) 150 bp (T→C) 167 bp (G→C) 273 bp (C→T)	Specific to ToxoDB#18, #29, #113 variants^c^
*SAG3*	Identical	Non-restriction site	3	89 bp (G→T) 90 bp (G→A) 110 bp (A→G)	True intra-genotypic diversity within ToxoDB#9
*BTUB*	Identical	Non-restriction site	1	145 bp (G→C)	Distinguishes ToxoDB#9 from reference Type Ⅱ
*GRA6*	Identical	Non-restriction site	1	195 bp (C→T)	Minor variation separating lineages

Abbreviations: SNP, single nucleotide polymorphism; PFLP, restriction fragment length polymorphism; *PK1*, protein kinase 1; *SAG3*, surface antigen 3; *BTUB*, beta-tubulin; *GRA6*, dense granule antigen 6.

Notes:

a All SNPs listed occur outside of the standard restriction enzyme recognition sites used in PCR-RFLP, making them invisible to traditional genotyping.

b Exact nucleotide positions correspond to the alignment files generated in this study.

c C22-8 variations were specific to certain non-ToxoDB#9 genotypes but highlight the diversity within the broader Type Ⅱ clade.

**Fig. 2:**
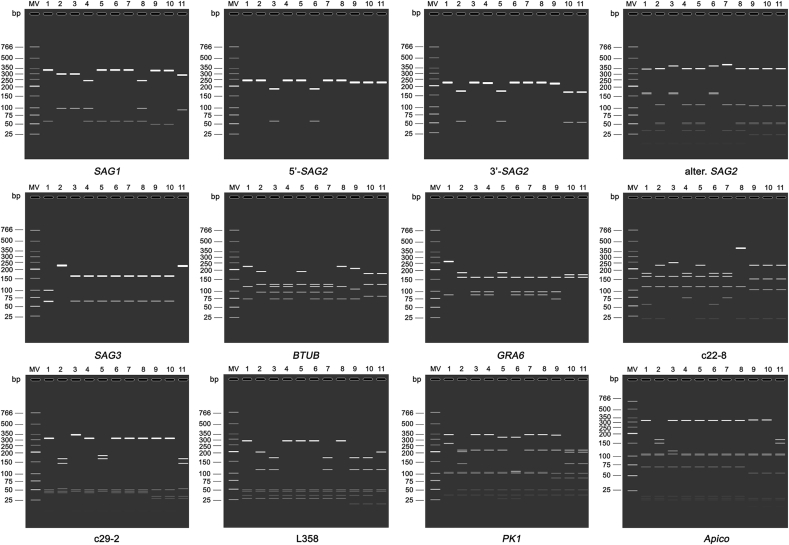
Diagrams showing *in silico* RFLP analyses of four *Toxoplasma gondii* isolates re-classified based on 12 loci. Lanes 1–12: GT1, PTG, CTG, MAS, TgCgCa1, TgCatBr5, TgCatBr64, TgRsCr1, TgC1, TgC2 and TgCYn14, respectively. MV represents the molecular weight marker. Abbreviations: *SAG1*, surface antigen 1; *SAG2*, surface antigen 2; *SAG3*, surface antigen 3; *BTUB*, beta-tubulin; *GRA6*, dense granule antigen 6; *PK1*, protein kinase 1.

Among these loci, *PK1* proved the most informative for resolving additional cryptic variation within Chinese *T. gondii* populations (Fig. S1). Across the dataset, 18 inter-genotypic and 4 intra-genotypic SNPs were detected. These were undetectable by conventional restriction enzyme-based methods. A deletion in the *PK1* sequence also served as a reliable molecular signature for all Type Ⅲ strains. Furthermore, high-resolution sequencing corrected the classification of three isolates: initially identified as ToxoDB#9 by conventional PCR-RFLP profiles, they were unequivocally re-assigned to distinct genotypes (ToxoDB#29, ToxoDB#1, and ToxoDB#113) (Fig. 2 and Table S3). Importantly, true intra-genotypic diversity was evident within the ostensibly monolithic ToxoDB#9 lineage. Non-synonymous SNPs at the *SAG3* locus, along with specific variations in C22-8 observed in variant genotypes (ToxoDB#18, ToxoDB#29, and ToxoDB#113), subdivided the clade into distinct genetic subgroups (Table 1). These findings indicate ongoing micro-evolutionary divergence and demonstrate the enhanced resolution provided by MLST compared to conventional RFLP.

### 3.3 Phylogenetic placement of Chinese ToxoDB#5 and ToxoDB#9 isolates

**Fig. 3:**
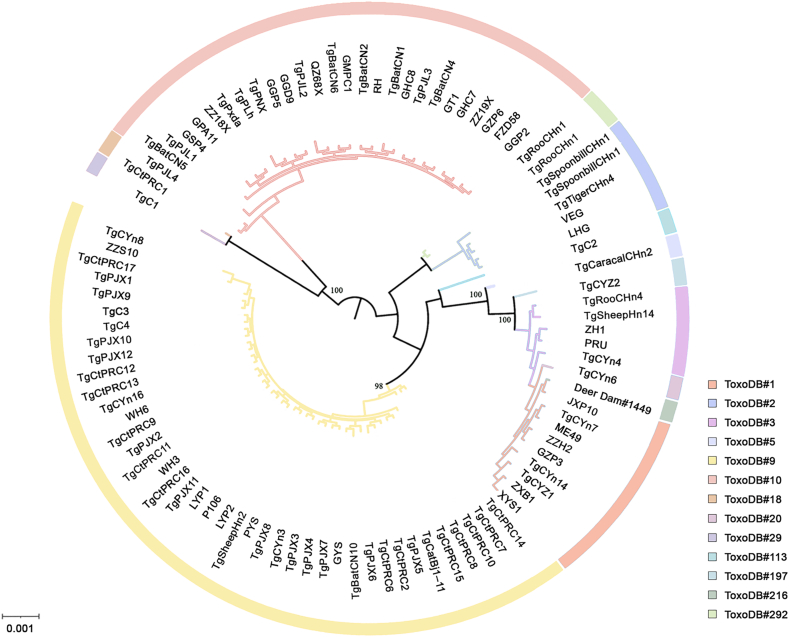
Maximum likelihood phylogenetic tree of *Toxoplasma gondii* based on concatenated sequences of 16 genetic loci (∼7062 bp). Bootstrap values are indicated at major nodes, illustrating the evolutionary relationships among Chinese isolates and reference strains.

The concatenated alignment of all 16 loci generated a dataset of 7062 bp per isolate. Phylogenetic analyses based on this concatenated dataset clarified the evolutionary relationships of the Chinese isolates (Fig. 3). The dominant ToxoDB#9 isolates formed a strongly supported monophyletic clade (Bootstrap = 98%) that clustered as a sister group to the archetypal Type Ⅱ lineage (e.g., ME49), confirming its classification as a distinct genotype closely related to Type Ⅱ lineages. Crucially, the ToxoDB#5 isolate (TgCaracalCHn2) was unambiguously placed within the phylogeny, receiving 100% bootstrap support and remaining clearly distinct from all Asian lineages (Fig. 3).

### 3.4 Haplotype expansion and lack of phylogeographic structure

**Fig. 4:**
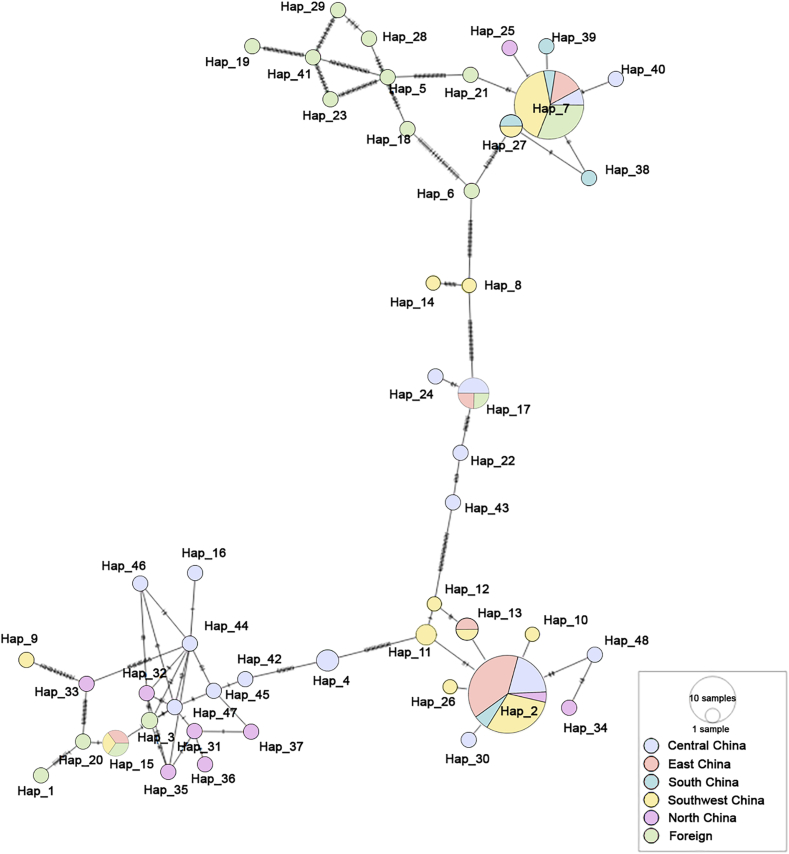
Minimum spanning network of *Toxoplasma gondii* haplotypes derived from concatenated 16-locus sequences. Node sizes correspond to haplotype frequency, and colors represent geographic origin of isolates. Star-like topology indicates central high-frequency haplotypes and peripheral low-frequency derivatives.

Among the 91 Chinese isolates (excluding five reference strains), we identified 36 unique haplotypes. Overall Hd and π across the five Chinese populations were 0.859 and 0.00379, respectively (Table 2). Hd ranged from 0.679 to 1.000, and π ranged from 0.00194 to 0.00409. Interestingly, the Southwest China population exhibited the highest Hd, whereas the East China population showed the lowest (Table 2). Including the five reference isolates increased the total to 48 unique haplotypes among all 96 sequenced isolates (Fig. S2). The minimum spanning network (MSN) constructed from the concatenated 16-locus sequences (7062 bp) revealed a star-like topology centered on two high-frequency haplotypes, Hap_2 (*n* = 29) and Hap_7 (*n* = 21), from which multiple low-frequency derivatives radiated (Fig. 4). Haplotypes from the six geographic populations were not segregated into independent branches; instead, they clustered around these central haplotypes, demonstrating a lack of clear phylogeographic structure. For example, Hap_2 was shared between Central and East China populations, while Hap_27 occurred in both North and South China populations (Fig. 4).

**Table 2:** Genetic diversity parameters based on combined sequence.

Geographical group	Number of samples	Percentage of total samples (%)	Number of haplotypes	Haplotype diversity (Hd)	Nucleotide diversity (π)	Parsimony informative sites
Central China	37	40.7	22	0.866	0.00338	82
East China	8	8.8	3	0.679	0.00409	44
South China	29	31.9	10	0.773	0.00352	61
Southwest China	11	12.1	11	1.000	0.00194	26
North China	6	6.6	5	0.933	0.00347	44
Total	91	100.0	41	0.859	0.00379	86

**Fig. 5:**
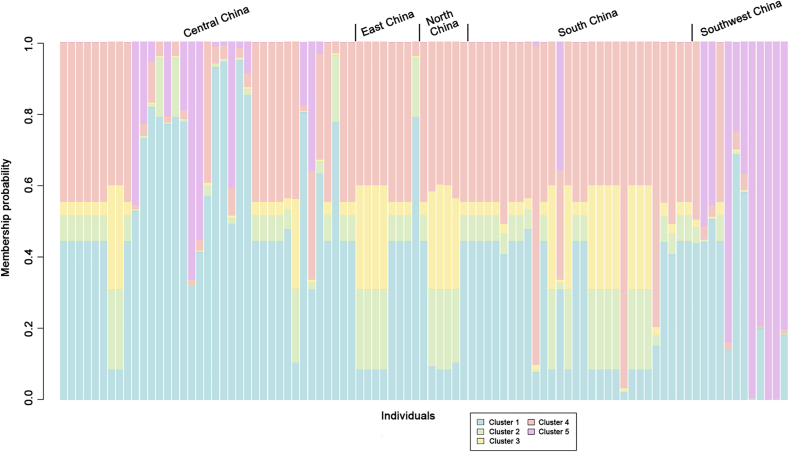
Genetic clustering of *Toxoplasma gondii* populations from China using the discriminant analysis of principal components (DAPC). Individual strains are aligned along the x-axis and grouped according to the region of origin. Strains are assigned either to one cluster (each cluster is represented by a different color) or to multiple clusters if their genotypes were admixed (indicated by multiple colors).

Based on the lowest BIC value, the DAPC optimally partitioned the dataset into five distinct genetic clusters (*K* = 5) (Fig. S3). The scatterplot based on the first two discriminant axes illustrated the extent of population segregation, showing the distribution and genetic overlap among these five clusters (Fig. S4). Furthermore, the DAPC optimally partitioned the dataset into five distinct genetic clusters (*K* = 5) (Fig. 5). The membership probability plot revealed a lack of strict geographic isolation. While some regional clustering was observed, most geographical regions, particularly Central China and South China, contained individuals assigned to multiple divergent clusters. The widespread sharing of genetic clusters across distant provinces is consistent with broad dispersal of clonal lineages through the movement of hosts and agricultural products, resulting in limited geographic structuring within this predominantly clonal lineage.

### 3.5 Genetic differentiation and genetic distance

**Fig. 6:**
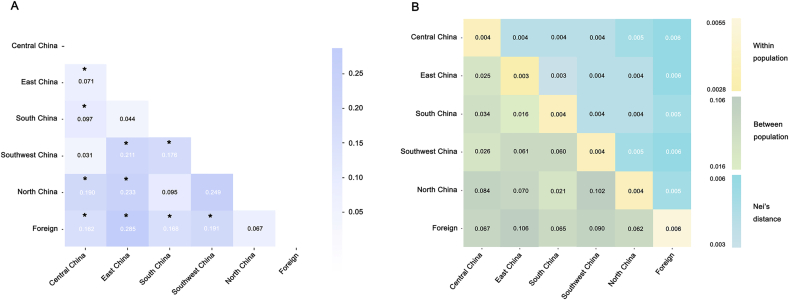
Genetic differentiation and distance among *Toxoplasma gondii* populations. A: Pairwise *F*_ST_ values among five Chinese geographic populations and the foreign group, illustrating the degree of genetic differentiation. B: Nei's genetic distance matrix among the same populations, highlighting relative genetic divergence between domestic and foreign isolates. The asterisk in panel A indicates *P* < 0.05.

Pairwise *F*_ST_ values among all populations ranged from 0.031 to 0.285 (Fig. 6A). Consistent with these metrics, *F*_ST_ values between the foreign group and domestic Chinese populations were substantially higher than those observed among domestic populations. The greatest differentiation was observed between the Foreign and East China groups (*F*_ST_ = 0.285), while the lowest involving the Foreign group was with North China (*F*_ST_ = 0.067) (Fig. 6A). Among the five domestic populations, the lowest differentiation occurred between Central China and Southwest China (*F*_ST_ = 0.031). With the exception of the Central–Southwest and East–South comparisons, all other domestic pairwise *F*_ST_ values ranged between 0.04 and 0.25, indicating moderate genetic differentiation. Nei's genetic distances among populations ranged from 0.003 to 0.006 (Fig. 6B). Both Nei's distances and mean K2P genetic distances between the Foreign group and the five domestic populations were noticeably higher than those observed among domestic populations. Within the domestic populations, the Central, South, East, and Southwest China groups exhibited the highest intra-population K2P distance, each recording a value of 0.004 (Fig. 6B), reflecting subtle but detectable intra-population variation.

AMOVA performed exclusively on ToxoDB#9 isolates (*n* = 41) revealed that 84.0% of the genetic variation resided within populations, while only 16.0% occurred among geographic groups, indicating substantial intra-genotypic diversity at the local level. The moderate *F*_ST_ value (0.137; *P* < 0.05) suggests high levels of gene flow or recent shared ancestry among ToxoDB#9 populations across the sampled regions.

## 4 Discussion

In this study, we integrated high-resolution MLST with conventional genotyping to overcome the long-standing “resolution ceiling” that has obscured the fine-scale epidemiology of *T*. *gondii* in China. By moving beyond the constraints of restriction-site analysis, we revealed substantial genetic diversity within the predominant ToxoDB#9 lineage and, notably, detected the ToxoDB#5 lineage (a lineage most frequently reported from North America) in a captive caracal, expanding its known geographic range. These findings are consistent with the concept of a dynamic global population structure [1,15]. However, whether the presence of ToxoDB#5 results from recent anthropogenic dissemination or reflects the detection of a rare, historically endemic lineage maintained in sylvatic cycles remains to be determined. Taken together, these findings highlight the value of continued molecular monitoring within a One Health framework.

The uncovering of cryptic diversity within ToxoDB#9 offers a molecular resolution to the “genotype-phenotype paradox” frequently reported in East Asia. While prior RFLP-based surveys suggested clonal expansion of a single genotype [26], our MLST data show that isolates with identical conventional genotypes can harbor fixed, non-synonymous SNPs at virulence-associated loci, such as *GRA6* and *PK1* [13,27]. These intra-genotypic polymorphisms likely explain the observed variation in murine virulence and may act as a reservoir of adaptive potential, enabling fine-tuned host–pathogen interactions. While previous studies have shown that epigenetic mechanisms, such as chromatin remodeling, can transiently modulate parasite division and transcriptional programs [13], the SNPs identified in our study represent stable genetic divergence. Together, both genetic and epigenetic variations support the view that ToxoDB#9 represents a micro-evolutionary complex rather than a monolithic clone [13]. This concept is further supported by previous phylogenetic and virulence divergence analyses, which revealed significant biological and genetic sub-structuring among Chinese ToxoDB#9 isolates [28], as well as by recent WGS analyses that demonstrated distinct ancestry compositions among different ToxoDB#9 isolates and detected historical recombination events within this lineage [16]. Consequently, reliance on RFLP alone underestimates the genetic diversity of *T. gondii* and may mask strain-to-strain variation in experimentally measured traits (e.g., murine virulence). Importantly, murine virulence is not necessarily predictive of clinical severity in immunocompetent humans, as some lineages can be highly lethal in mice yet commonly cause asymptomatic infections in people, such as ToxoDB#6 (Africa 1). Similar cryptic intra-genotypic diversity has been reported in European and North American populations [3], suggesting that our observations are not unique to China but reflect a broader evolutionary phenomenon.

A notable finding of this study is the detection of ToxoDB#5 (HG12) genotype in a captive caracal (*C*. *caracal*) in China. This genotype is frequently reported in North American sylvatic cycles, where it is common in wildlife and in grazing livestock that may encounter wildlife-contaminated environments (e.g., sheep), while remaining very rare in strictly confined domestic animals [3,9,29]. Recent genotyping of human toxoplasmosis cases in North America revealed that HG12 strains, including ARI and RAY, are not only prevalent in wildlife but also common in humans in that region [30]. It is noteworthy that the two historically well-characterized ToxoDB#5 strains were isolated from patients in the United States [30]. In addition, a study reported symptomatic toxoplasmosis in immunocompetent individuals following the consumption of game meat contaminated with HG12 strains, although clinical manifestations were milder than those associated with highly virulent Amazonian strains [31]. However, the generalizability of these findings should be interpreted with caution, as the source of infection was unique and the infectious dose was unknown. An experimental study has demonstrated that strains causing asymptomatic infections at low or moderate doses may become lethal at higher doses [32]. Whether HG12 strains are more pathogenic to humans than Type Ⅱ remains to be clarified [30]. Accordingly, their public health implications warrant further investigation rather than immediate concern. Notably, ToxoDB#5 has also been reported in marine hosts in China [33], suggesting that detections in China may not be limited to the present study and warrant broader investigation across terrestrial and marine interfaces.

The presence of ToxoDB#5 in China can be explained by at least two non-mutually exclusive scenarios. One possibility is anthropogenic introduction, such as through the translocation of zoo animals, which would align with the concept of “pathogen pollution” [7,34]. Alternatively, and perhaps more parsimoniously, this genotype may represent a rare, historically endemic lineage maintained in sylvatic cycles that has previously gone undetected. The existence of genotypes largely restricted to wildlife has been linked to the presence of substantial wild felid populations, as documented in North America and certain regions of South America and Africa [3,9,35,36]. In North America, ToxoDB#5 has not successfully established itself within domestic transmission cycles, likely due to limited adaptation to domestic cats and domestic environments [37,38]. Its detection in grazing livestock in that region is consistent with the encroachment of livestock into habitats occupied by wild felids, leading to incidental exposure to oocysts of wildlife-associated strains, rather than sustained domestic transmission [37]. In China, where wild felid populations have severely declined, wildlife-associated genotypes such as ToxoDB#5 would be expected to be extremely rare, making their detection inherently difficult and consistent with low-level endemicity rather than recent introduction. Regardless of its origin, the detection of a divergent lineage within the Chinese *T. gondii* population highlights the value of continued molecular surveillance [39,40].

Our MLST-based population genetic analyses further indicate that 84.0% of genetic variance within ToxoDB#9 resides within populations (AMOVA), supported by a star-like haplotype network centered on dominant haplotypes (Hap_2 and Hap_7). Importantly, the high haplotype diversity observed within ToxoDB#9 should not be interpreted as evidence of a panmictic population structure or frequent sexual recombination. Rather, this diversity is consistent with the accumulation of mutations over long evolutionary timescales within a predominantly clonal lineage. An analogous pattern has been documented in the Type Ⅱ lineage globally: microsatellite-based analyses revealed relatively high intra-lineage diversity [41], yet WGS of dozens of Type Ⅱ strains from multiple continents unequivocally demonstrated that this diversity arose through mutation under strict clonality [38]. The absence of isolation-by-distance is consistent with human-mediated dissemination of prevalent clonal lineages, a pattern previously reported in other regions [[42], [43], [44]]. In our context, the unrestricted movement of food animals appears to be the primary driver facilitating the dispersal dominant clones (ToxoDB#9). While sexual recombination between lineages is generally rare, the detection of divergent lineages (e.g., ToxoDB#5) in the same geographical areas raises the theoretical possibility of coinfection in definitive hosts, which is a necessary prerequisite for the generation of novel recombinant genotypes [[45], [46], [47]]. However, this remains a low-probability scenario under the predominantly clonal reproductive mode of *T. gondii* and cannot be evaluated with our MLST dataset.

From a practical standpoint, these findings underscore the need for genomic-scale monitoring. WGS-based monitoring could detect potential introductions, characterize microevolutionary changes at loci implicated in virulence, and evaluate the genomic signatures of recombinant co-circulating lineages. While MLST is a substantial improvement over RFLP, it lacks the full resolution of WGS, limiting precise reconstruction of ToxoDB#5 introduction timelines and mutation rates. Moreover, the phenotypic impact of the identified SNPs remains to be confirmed through functional validation [6]. Because murine virulence does not reliably predict clinical outcomes in humans, any *in vivo* assays should be interpreted as lineage-specific measures of pathogenicity rather than direct proxies for human disease. The current sample size for exotic lineages is small, and broader, systematic molecular monitoring is required to determine whether ToxoDB#5 has established a sustainable cycle in China or represents sporadic introductions.

Our MLST results generate testable hypotheses for future work. First, future WGS of Chinese ToxoDB#5 isolate, along with any additional isolates identified through expanded surveillance, could help elucidate its geographic origins and evolutionary history. However, because HG12 is not a strictly clonal lineage and exhibits substantial genetic diversity among strains in North America, interpreting genome-wide divergence patterns will require comparison against a broad panel of geographically diverse HG12 reference genomes rather than a single representative strain. Reduced diversity relative to the North American source population, or phylogenetic nesting within a specific North American sub-lineage, would support a recent founder introduction, whereas diversity levels comparable to North American populations could indicate longer-term cryptic circulation or multiple independent introductions. Second, if the cryptic SNP-defined subgroups within ToxoDB#9 reflect distinct clonal lineages, then genome-wide linkage disequilibrium and ancestry blocks should support at least two major clonal backgrounds with limited recent recombination, and these backgrounds may correlate with variation in parasite replication/virulence phenotypes in standardized mouse or cell-culture assays.

## 5 Conclusion

This study redefines the phylogeography of *T. gondii* in China by applying MLST to resolve within-genotype diversity and geographic distribution patterns that are not captured by RFLP-based typing. Our data indicates the co-circulation of multiple genotypes, including rare lineages detected in limited samples; however, MLST alone does not allow robust inference on the timing, directionality, or drivers of their movement. Risk assessment and source attribution for toxoplasmosis will benefit from systematic molecular monitoring within a One Health framework [48], coupled where feasible with WGS to (1) confirm introductions, (2) better resolve relatedness among isolates, and (3) assess the potential for recombination where distinct lineages co-occur. Overall, our results provide an updated baseline for future, larger-scale sampling and risk assessment across human, livestock, wildlife, and environmental interfaces.

## CRediT authorship contribution statement

**Yi-Tian Fu:** Writing – review & editing, Writing – original draft, Investigation, Formal analysis, Data curation, Conceptualization. **Yu-Na Xiao:** Investigation, Formal analysis. **Xi-Long Yi:** Investigation, Formal analysis, Conceptualization. **Hany M. Elsheikha:** Writing – review & editing, Conceptualization. **Bei-Bei Zhou:** Investigation, Formal analysis. **Meng-Ling Deng:** Investigation, Formal analysis. **Feng-Cai Zou:** Investigation, Funding acquisition. **Xing-Quan Zhu:** Writing – review & editing, Supervision, Resources, Project administration, Funding acquisition, Conceptualization. **Guo-Hua Liu:** Writing – review & editing, Writing – original draft, Supervision, Resources, Project administration, Methodology, Funding acquisition, Conceptualization.

## Ethics approval and consent to participate

Not applicable.

## Availability of data and materials

The newly acquired sequences of *Toxoplasmagondii* have been deposited in the GenBank database under the accession numbers PV191327–PV192891.

## Funding statement

This work was supported by the NSFC-Yunnan Joint Fund (grant number U2202201), the 10.13039/501100012166National Key Research and Development Program of China (grant numbers 2021YFC2300800 and 2021YFC2300802), the Science and Technology Innovation Program of Hunan Province (grant number 2025RC1053), and the Special Research Fund of Shanxi Agricultural University for High-level Talents (grant number 2021XG001).

## Declaration of competing interests

The authors declare the following financial interests/personal relationships which may be considered as potential competing interests:

Given his role as an associate editor for the journal *Science in One Health*, and given his role as a guest editor for the special issue “Surveillance and Response to Emerging and Re-emerging Zoonotic Diseases”, Prof Xing-Quan Zhu had no involvement in the peer review of this article and had no access to information regarding its peer review. Full responsibility for the editorial process for this article was delegated to another journal editor. If there are other authors, they declare that they have no known competing financial interests or personal relationships that could have appeared to influence the work reported in this paper.
