# Supplementary figures and images for: Population genetic structure of zoonotic Toxoplasma gondii in China revealed using multilocus sequence typing
Source: Sci One Health. 2026 May 27;5:100162. doi: 10.1016/j.soh.2026.100162 (PMC13276426; doi:10.1016/j.soh.2026.100162)

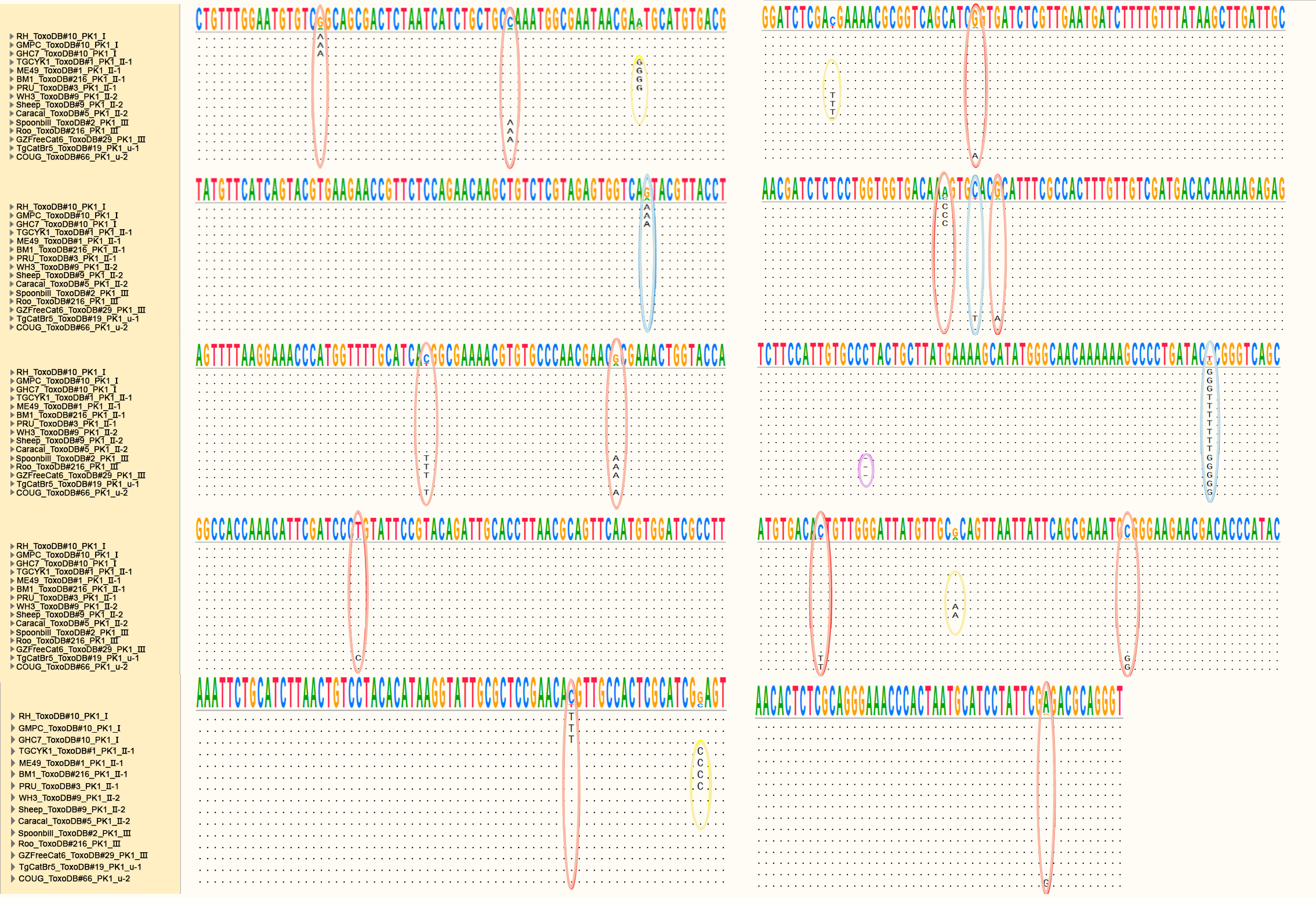

Supplement: Multimedia component 1 [file mmc1.zip › Fig. S1.tif]

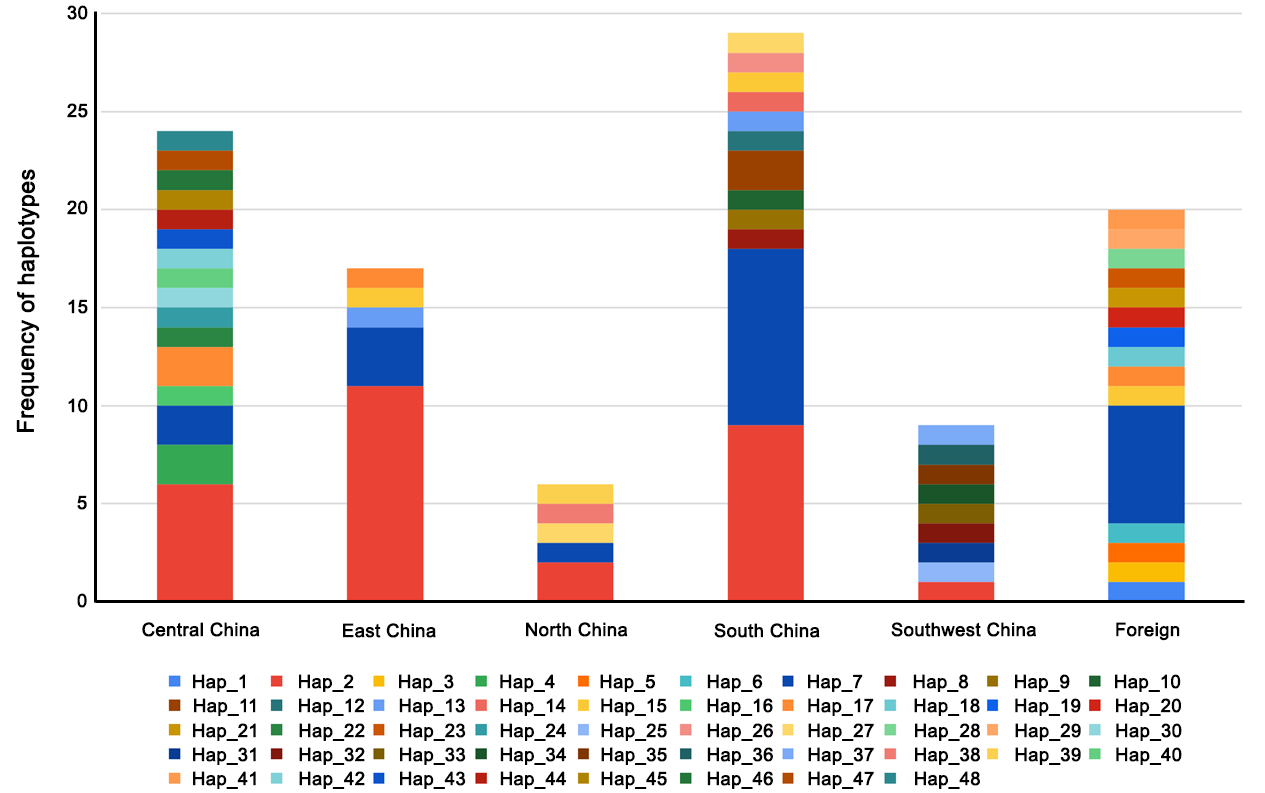

Supplement: Multimedia component 1 [file mmc1.zip › Fig. S2.tif]

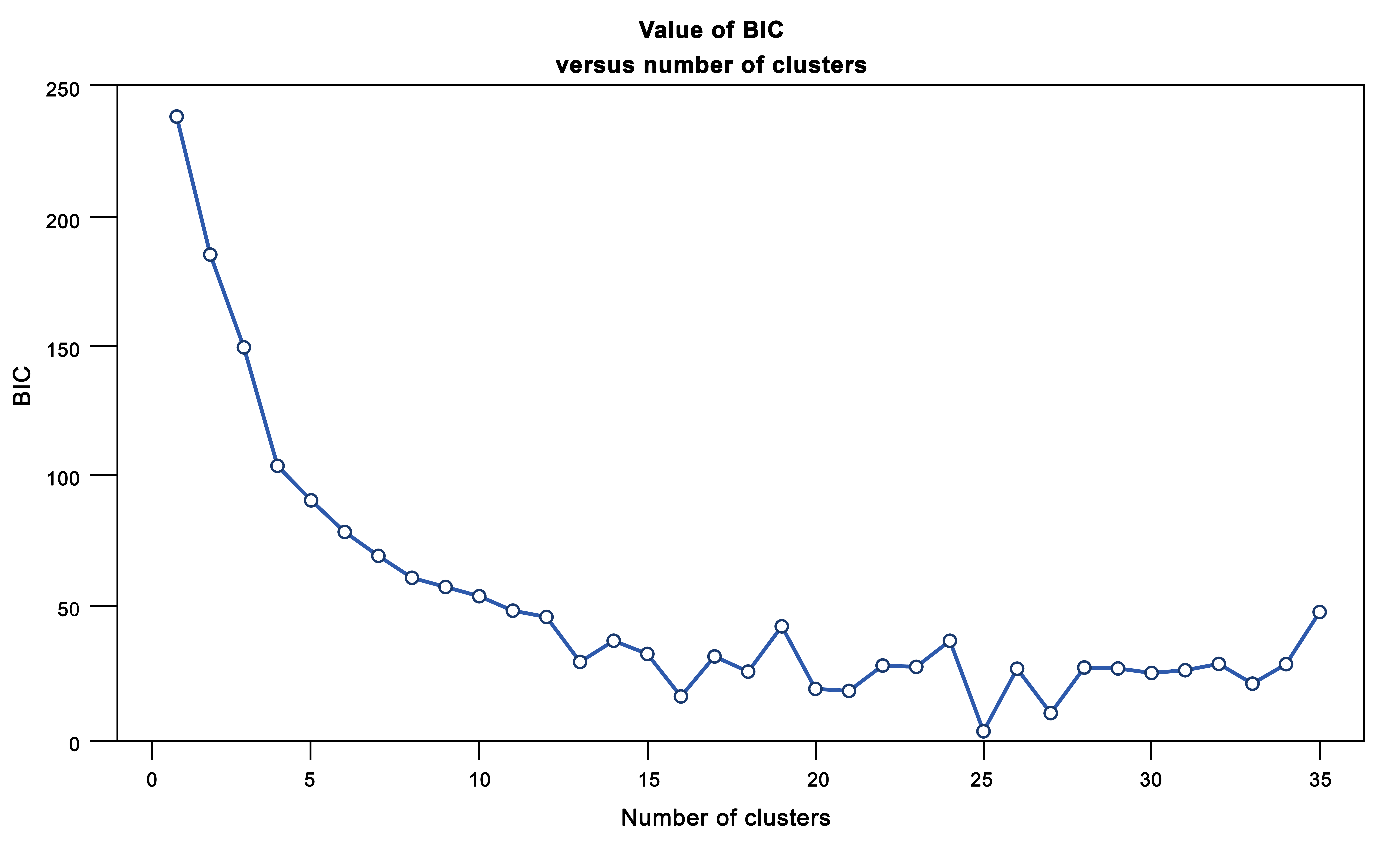

Supplement: Multimedia component 1 [file mmc1.zip › Fig. S3.tif]

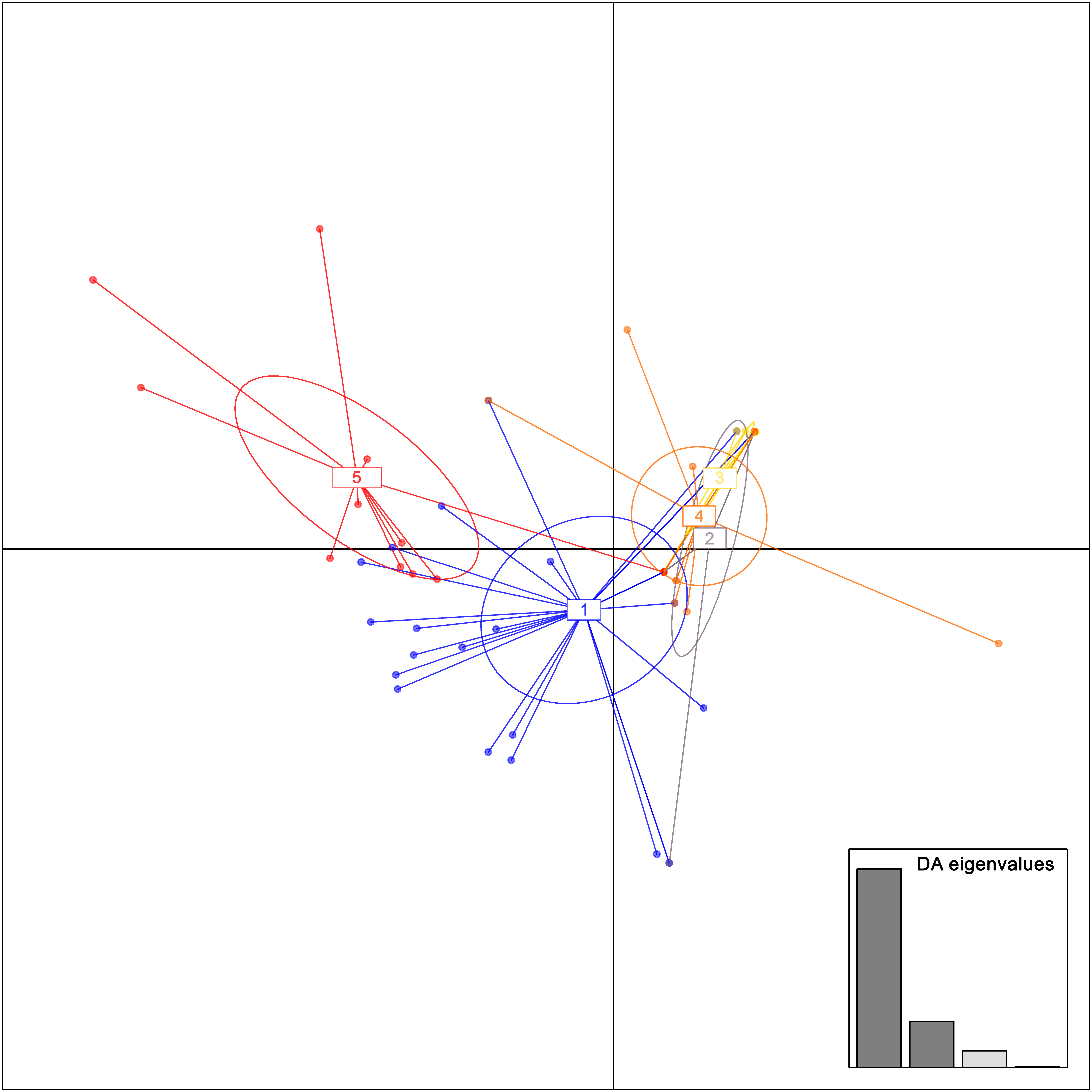

Supplement: Multimedia component 1 [file mmc1.zip › Fig. S4.tif]
