# Supplementary material for: Population genetic structure of zoonotic Toxoplasma gondii in China revealed using multilocus sequence typing
Source: Sci One Health. 2026 May 27;5:100162. doi: 10.1016/j.soh.2026.100162 (PMC13276426; doi:10.1016/j.soh.2026.100162)
Supplement: Multimedia component 2 [file mmc2.docx]

**Supplementary Materials**

**Supplementary Figures**


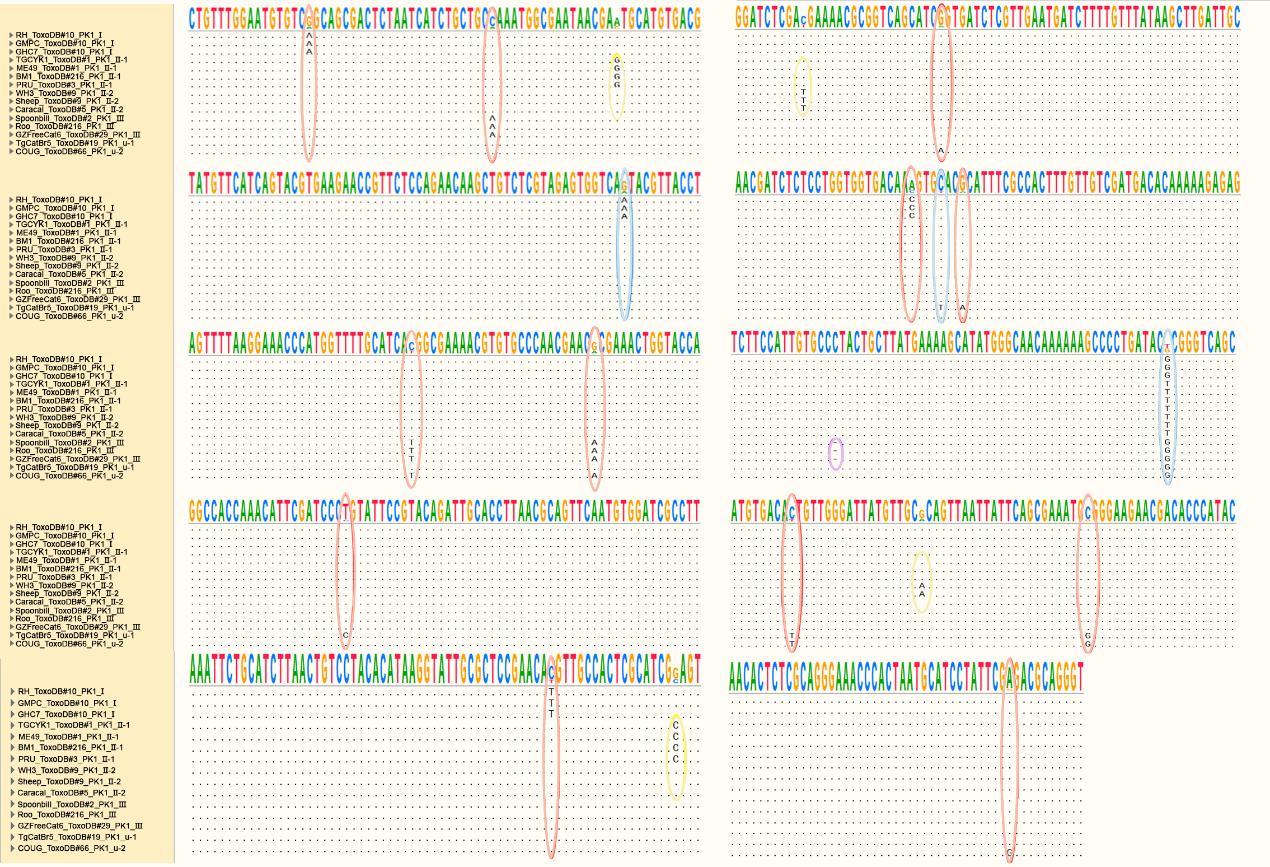


**Fig. S1.** Scatterplot indicating axes 1 and 2 of the discriminant analysis of principal component analysis (PCA)-transformed data (DAPC). Individual clones are indicated by dots. Numbers and colors mention the five genetic clusters retained from Bayesian Information Criterion (BIC) values.


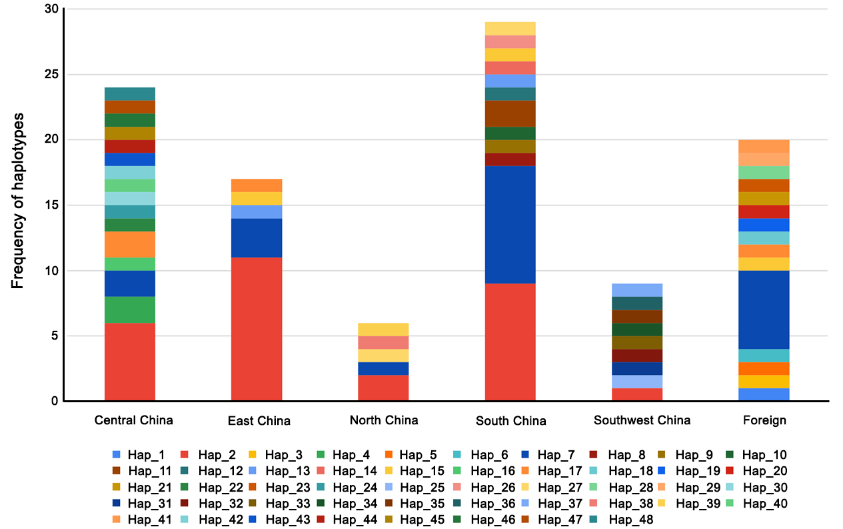


**Fig. S2.** Genetic clustering of *Toxoplasma gondii* populations from China using the discriminant analysis of principal components (DAPC). Bayesian Information Criterion (BIC) is provided for different numbers of clusters (from 1 to 35).


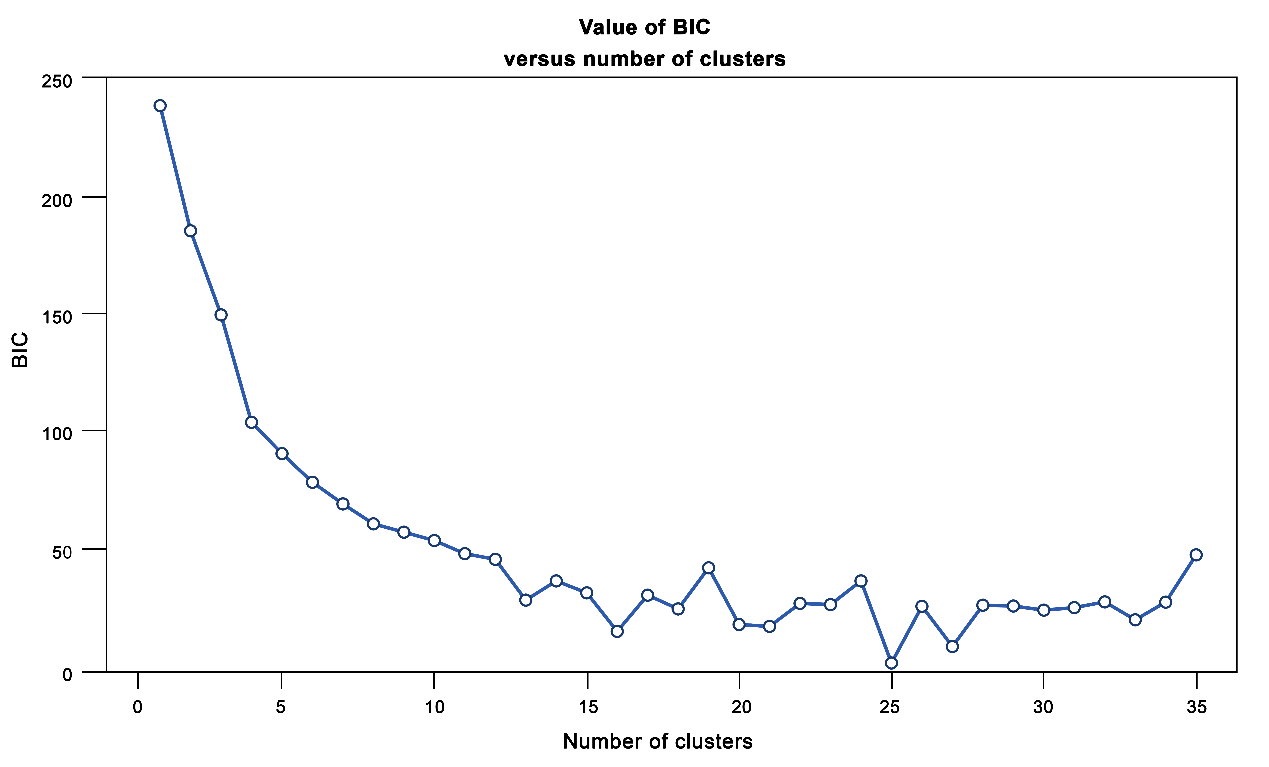


**Fig. S3.** Geographic distribution of *Toxoplasma gondii* haplotypes in China, illustrating the prevalence of dominant and rare haplotypes across sampled provinces. Abbreviation: BIC, Bayesian Information Criterion.


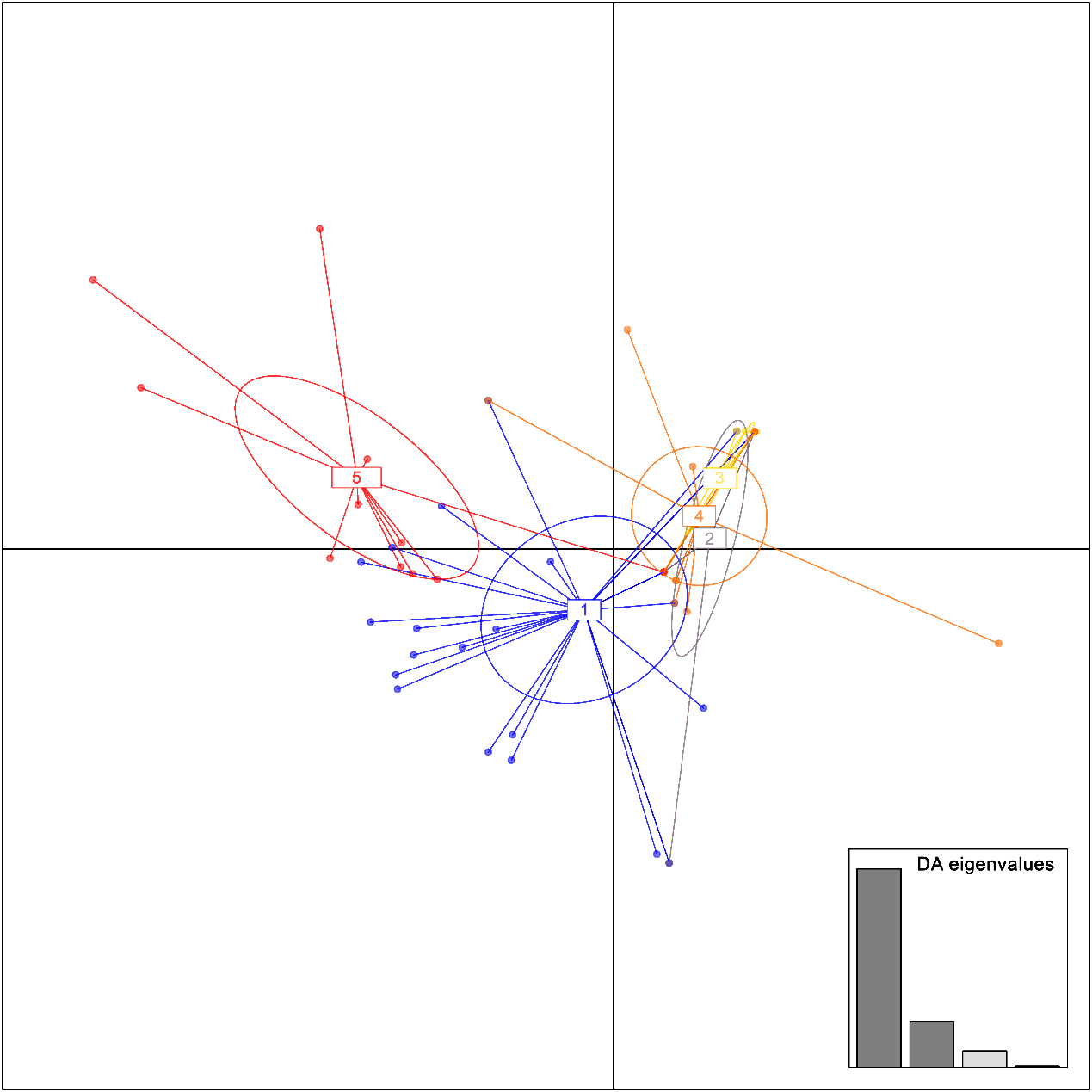


**Fig. S4.** Nucleotide sequence alignment of the *PK1* gene across distinct *Toxoplasma gondii* genotypes, highlighting intra- and inter-genotypic polymorphisms. Abbreviations: DA, discriminant analysis; *PK1*, protein kinase 1.

**Supplementary Tables**

**Table S1.** *Toxoplasma gondii* isolates analyzed in this study, their host, sampling location, and genotypes.

| Isolate ID | Host | Sampling location | Genotype (previous study) | Genotype (this study) | Note | Reference |
| --- | --- | --- | --- | --- | --- | --- |
| TgBatCN6 | Black-bearded tomb bat | Unknown, Guangxi | Unknown | ToxoDB#10 | First genotype identification | Unpublished |
| Deer Dam#1449 | Brazilian Elk | A Zoo in Brazil | ToxoDB#216 | ToxoDB#216 | Exotic host | [1] |
| TgCaracalCHn2 | Caracal | A Zoo in Henan | ToxoDB#5 | ToxoDB#5 | Exotic host; Novel lineage | [2] |
| TgCatBj1–11 | Cat | Unknown, Beijing | ToxoDB#9 | ToxoDB#9 | No change | [3] |
| TgC1 | Cat | Guangzhou, Guangdong | ToxoDB#9 | ToxoDB#29 | Genotype change | [4] |
| TgC2 | Cat | Guangzhou, Guangdong | ToxoDB#9 | ToxoDB#113 | Genotype change | [4] |
| TgC3 | Cat | Guangzhou, Guangdong | ToxoDB#9 | ToxoDB#9 | No change | [4] |
| TgC4 | Cat | Guangzhou, Guangdong | ToxoDB#9 | ToxoDB#9 | No change | [4] |
| TgCtPRC1 | Cat | Guangzhou, Guangdong | ToxoDB#18 | ToxoDB#18 | No change | [5] |
| TgCtPRC2 | Cat | Guangzhou, Guangdong | ToxoDB#9 | ToxoDB#9 | No change | [5] |
| TgCtPRC6 | Cat | Guangzhou, Guangdong | ToxoDB#9 | ToxoDB#9 | No change | [5] |
| TgCtPRC7 | Cat | Guangzhou, Guangdong | ToxoDB#9 | ToxoDB#9 | No change | [5] |
| TgCtPRC8 | Cat | Guangzhou, Guangdong | ToxoDB#9 | ToxoDB#9 | No change | [5] |
| TgCtPRC9 | Cat | Guangzhou, Guangdong | ToxoDB#9 | ToxoDB#9 | No change | [5] |
| TgCtPRC10 | Cat | Guangzhou, Guangdong | ToxoDB#9 | ToxoDB#9 | No change | [5] |
| TgCtPRC11 | Cat | Guangzhou, Guangdong | ToxoDB#9 | ToxoDB#9 | No change | [5] |
| TgCtPRC12 | Cat | Guangzhou, Guangdong | ToxoDB#9 | ToxoDB#9 | No change | [5] |
| TgCtPRC13 | Cat | Guangzhou, Guangdong | ToxoDB#9 | ToxoDB#9 | No change | [5] |
| TgCtPRC14 | Cat | Guangzhou, Guangdong | ToxoDB#9 | ToxoDB#9 | No change | [5] |
| TgCtPRC15 | Cat | Guangzhou, Guangdong | ToxoDB#9 | ToxoDB#9 | No change | [5] |
| TgCtPRC16 | Cat | Guangzhou, Guangdong | ToxoDB#9 | ToxoDB#9 | No change | [5] |
| TgCtPRC17 | Cat | Guangzhou, Guangdong | ToxoDB#9 | ToxoDB#9 | No change | [5] |
| GHC7 | Cat | Heyuan, Guangdong | Unknown | ToxoDB#10 | First genotype identification | Unpublished |
| GHC8 | Cat | Heyuan, Guangdong | Unknown | ToxoDB#10 | First genotype identification | Unpublished |
| TgCYn3 | Cat | Kunming, Yunnan | ToxoDB#9 | ToxoDB#9 | No change | [6] |
| TgCYn4 | Cat | Yimen, Yunnan | ToxoDB#3 | ToxoDB#3 | No change | [6] |
| TgCYn6 | Cat | Jinping, Yunnan | ToxoDB #20 | ToxoDB#20 | No change | [6] |
| TgCYn7 | Cat | Jinping, Yunnan | ToxoDB#1 | ToxoDB#1 | No change | [6] |
| TgCYn8 | Cat | Jinping, Yunnan | ToxoDB#9 | ToxoDB#9 | No change | [6] |
| TgCYn14 | Cat | Jinping, Yunnan | ToxoDB#9 | ToxoDB#1 | Genotype change | [6] |
| TgCYn16 | Cat | Jinping, Yunnan | ToxoDB#9 | ToxoDB#9 | No change | [6] |
| TgCYZ1 | Cat | Kunming, Yunnan | Unknown | ToxoDB#1 | First genotype identification | Unpublished |
| TgCYZ2 | Cat | Yuxi, Yunnan | Unknown | ToxoDB#197 | First genotype identification | Unpublished |
| WH3 | Cat | Wuhan, Hubei | ToxoDB#9 | ToxoDB#9 | No change | [7] |
| WH6 | Cat | Wuhan, Hubei | ToxoDB#9 | ToxoDB#9 | No change | [7] |
| QZ68X | Chicken | Quanzhou, Fujian | ToxoDB#10 | ToxoDB#10 | No change | [8] |
| ZZ18X | Chicken | Zhangzhou, Fujian | ToxoDB#10 | ToxoDB#10 | No change | [8] |
| ZZ19X | Chicken | Zhangzhou, Fujian | ToxoDB#10 | ToxoDB#10 | No change | [8] |
| GGD9 | Dog | Guangzhou, Guangdong | Unknown | ToxoDB#10 | First genotype identification | Unpublished |
| FZD58 | Duck | Quanzhou, Fujian | ToxoDB#10 | ToxoDB#10 | No change | [9] |
| GT1 | Goat | USA | ToxoDB#10 | ToxoDB#10 | Reference strain | [10] |
| TgRooCHn1 | Grey Kangaroo | A Zoo in Henan | ToxoDB#292 | ToxoDB#292 | Exotic host | [11] |
| TgRooCHn1 | Grey Kangaroo | A Zoo in Henan | ToxoDB#292 | ToxoDB#292 | Exotic host | [11] |
| PRU | Human | France | ToxoDB#3 | ToxoDB#3 | Reference strain | [10] |
| LHG | Human | Hefei, Anhui | ToxoDB#2 | ToxoDB#2 | No change | Unpublished |
| RH | Human | USA | ToxoDB#10 | ToxoDB#10 | Reference strain | [10] |
| VEG | Human | USA | ToxoDB#2 | ToxoDB#2 | Reference strain | [10] |
| TgBatCN4 | Intermediate leaf-nosed bat | Unknown, Guangxi | ToxoDB#10 | ToxoDB#10 | No change | [12] |
| TgBatCN5 | Intermediate leaf-nosed bat | Unknown, Guangxi | ToxoDB#10 | ToxoDB#10 | No change | [12] |
| TgBatCN10 | Intermediate leaf-nosed bat | Unknown, Guangxi | ToxoDB#9 | ToxoDB#9 | No change | [12] |
| GMPC1 | Masked Palm Civet | Shaoguan, Guangdong | Unknown | ToxoDB#10 | First genotype identification | Unpublished |
| GPA11 | Parrot | Foshan, Guangdong | Unknown | ToxoDB#10 | First genotype identification | Unpublished |
| TgPNX | Pig | Neixiang, Henan | ToxoDB#10 | ToxoDB#10 | No change | [13] |
| TgPxda | Pig | Luohe, Henan | ToxoDB#10 | ToxoDB#10 | No change | [13] |
| TgPLh | Pig | Xinyang, Henan | ToxoDB#10 | ToxoDB#10 | No change | [13] |
| GGP2 | Pig | Guangzhou, Guangdong | Unknown | ToxoDB#10 | First genotype identification | Unpublished |
| GGP5 | Pig | Guangzhou, Guangdong | Unknown | ToxoDB#10 | First genotype identification | Unpublished |
| PYS | Pig | Guangzhou, Guangdong | ToxoDB#9 | ToxoDB#9 | No change | [4] |
| GSP4 | Pig | Shenzhen, Guangdong | Unknown | ToxoDB#10 | First genotype identification | Unpublished |
| GZP3 | Pig | Zhongshan, Guangdong | Unknown | ToxoDB#1 | First genotype identification | Unpublished |
| GZP6 | Pig | Zhongshan, Guangdong | Unknown | ToxoDB#10 | First genotype identification | Unpublished |
| GYS | Pig | Baiyin, Gansu | ToxoDB#9 | ToxoDB#9 | No change | [4] |
| LYP1 | Pig | Longyan, Fujian | Unknown | ToxoDB#9 | First genotype identification | Unpublished |
| LYP2 | Pig | Longyan, Fujian | Unknown | ToxoDB#9 | First genotype identification | Unpublished |
| TgPJL1 | Pig | Jiutai, Jilin | ToxoDB#10 | ToxoDB#10 | No change | [14] |
| TgPJL2 | Pig | Jiutai, Jilin | ToxoDB#10 | ToxoDB#10 | No change | [14] |
| TgPJL3 | Pig | Meihekou, Jilin | ToxoDB#10 | ToxoDB#10 | No change | [14] |
| TgPJL4 | Pig | Unknown, Jilin | Unknown | ToxoDB#10 | First genotype identification | [14] |
| TgPJX1 | Pig | Wannian, Jiangxi | ToxoDB#9 | ToxoDB#9 | No change | [15] |
| TgPJX2 | Pig | Xinjian, Jiangxi | ToxoDB#9 | ToxoDB#9 | No change | [15] |
| TgPJX3 | Pig | Nanchang, Jiangxi | ToxoDB#9 | ToxoDB#9 | No change | [15] |
| TgPJX4 | Pig | Xingguo, Jiangxi | ToxoDB#9 | ToxoDB#9 | No change | [15] |
| TgPJX5 | Pig | Xingguo, Jiangxi | ToxoDB#9 | ToxoDB#9 | No change | [15] |
| TgPJX6 | Pig | Nanchang, Jiangxi | ToxoDB#9 | ToxoDB#9 | No change | [15] |
| TgPJX7 | Pig | Yujiang, Jiangxi | ToxoDB#9 | ToxoDB#9 | No change | [15] |
| TgPJX8 | Pig | Yujiang, Jiangxi | ToxoDB#9 | ToxoDB#9 | No change | [15] |
| TgPJX9 | Pig | Yujiang, Jiangxi | ToxoDB#9 | ToxoDB#9 | No change | [15] |
| TgPJX10 | Pig | Yujiang, Jiangxi | ToxoDB#9 | ToxoDB#9 | No change | [15] |
| TgPJX11 | Pig | Yujiang, Jiangxi | ToxoDB#9 | ToxoDB#9 | No change | [15] |
| TgPJX12 | Pig | Nanchang, Jiangxi | ToxoDB#9 | ToxoDB#9 | No change | [15] |
| JXP10 | Pig | Unknown, Jiangxi | Unknown | ToxoDB#1 | First genotype identification | Unpublished |
| P106 | Pig | Yangzhou, Jiangsu | ToxoDB#9 | ToxoDB#9 | No change | [16] |
| TgSheepHn14 | Sheep | Jiaozuo, Henan | ToxoDB#3 | ToxoDB#3 | No change | [17] |
| TgSheepHn2 | Sheep | Jiaozuo, Henan | ToxoDB#9 | ToxoDB#9 | No change | [18] |
| XYS1 | Sheep | Xinxiang, Henan | Unknown | ToxoDB#1 | First genotype identification | Unpublished |
| ZZS10 | Sheep | Zhengzhou, Henan | Unknown | ToxoDB#9 | First genotype identification | Unpublished |
| ME49 | Sheep | USA | ToxoDB#1 | ToxoDB#1 | Reference strain | [10] |
| TgTigerCHn4 | Siberian Tiger | A Zoo in Henan | ToxoDB#2 | ToxoDB#2 | No change | [19] |
| ZH1 | Sloth | A Zoo in Henan | Unknown | ToxoDB#3 | Exotic host | Unpublished |
| ZZH2 | Sloth | A Zoo in Henan | Unknown | ToxoDB#1 | Exotic host | Unpublished |
| TgSpoonbillCHn1 | Spoonbill | A Zoo in Henan | ToxoDB#2 | ToxoDB#2 | No change | [20] |
| TgSpoonbillCHn1 | Spoonbill | A Zoo in Henan | ToxoDB#2 | ToxoDB#2 | No change | [20] |
| ZXB1 | Spotted Hyena | A Zoo in Henan | Unknown | ToxoDB#1 | Exotic host | Unpublished |
| TgBatCN1 | Trident leaf-nosed bat | Unknown, Yunnan | ToxoDB#10 | ToxoDB#10 | No change | [12] |
| TgBatCN2 | Trident leaf-nosed bat | Unknown, Yunnan | ToxoDB#10 | ToxoDB#10 | No change | [12] |
| TgRooCHn4 | White Kangaroo | A Zoo in Henan | ToxoDB#3 | ToxoDB#3 | Exotic host | [21] |

**Table S2.** Primer sequences used for multiplex and nested PCR amplification of *Toxoplasma gondii* loci.

| Gene locus | Multiplex PCR outer primer (5'→3') | PCR inner primer (5'→3') | Amplified size (bp) |
| --- | --- | --- | --- |
| *SAG1* | F: GTTCTAACCACGCACCCTGAG  R: AAGAGTGGGAGGCTCTGTGA | F: CAATGTGCACCTGTAGGAAGC  R: GTGGTTCTCCGTCGGTGTGAG | 377 |
| 5'-*SAG2* | F: GGAACGCGAACAATGAGTTT  R: GCACTGTTGTCCAGGGTTTT | F: GAAATGTTTCAGGTTGCTGC  R: GCAAGAGCGAACTTGAACAC | 228 |
| 3'-*SAG2* | F: TCTGTTCTCCGAAGTGACTCC  R: TCAAAGCGTGCATTATCGC | F: ATTCTCATGCCTCCGCTTC  R: AACGTTTCACGAAGGCACAC | 216 |
| alter. *SAG2* | F: GGAACGCGAACAATGAGTTT  R: GCACTGTTGTCCAGGGTTTT | F: ACCCATCTGCGAAGAAAACG  R: ATTTCGACCAGCGGGAGCAC | 525 |
| *SAG3* | F: CAACTCTCACCATTCCACCC  R: GCGCGTTGTTAGACAAGACA | F: TCTTGTCGGGTGTTCACTCA  R: CACAAGGAGACCGAGAAGGA | 224 |
| *BTUB* | F: TCCAAAATGAGAGAAATCGT  R: AAATTGAAATGACGGAAGAA | F: GAGGTCATCTCGGACGAACA  R: TTGTAGGAACACCCGGACGC | 388 |
| *GRA6* | F: ATTTGTGTTTCCGAGCAGGT  R: GCACCTTCGCTTGTGGTT | F: TTTCCGAGCAGGTGACCT  R: TCGCCGAAGAGTTGACATAG | 331 |
| C22-8 | F: TGATGCATCCATGCGTTTAT  R: CCTCCACTTCTTCGGTCTCA | F: TCTCTCTACGTGGACGCC  R: AGGTGCTTGGATATTCGC | 496 |
| C29-2 | F: ACCCACTGAGCGAAAAGAAA  R: AGGGTCTCTTGCGCATACAT | F: AGTTCTGCAGAGTGTCGC  R: TGTCTAGGAAAGAGGCGC | 421 |
| L358 | F: TCTCTCGACTTCGCCTCTTC  R: GCAATTTCCTCGAAGACAGG | F: AGGAGGCGTAGCGCAAGT  R: CCCTCTGGCTGCAGTGCT | 394 |
| *PK1* | F: GAAAGCTGTCCACCCTGAAA  R: AGAAAGCTCCGTGCAGTGAT | F: CGCAAAGGGAGACAATCAGT  R: TCATCGCTGAATCTCATTGC | 820 |
| *Apico* | F: TGGTTTTAACCCTAGATTGTGG  R: AAACGGAATTAATGAGATTTGAA | F: GCAAATTCTTGAATTCTCAGTT  R: GGGATTCGAACCCTTGATA | 613 |
| *EF* | F: AAATGCACCCTTTTCTTAAA  R: CACATGAAGGTACACCAAAA | F: AAATTGTCCCGCCATCAG  R: CACATGAAGGTACACCAAAA | 530 |
| *HP* | F: GACAGAAACACGCAGAGAAT  R: TAATCTTTGTTCCCATGCTT | F: ATAATACAGTCAGTTCCCTCGAT  R: TAATCTTTGTTCCCATGCTT | 488 |
| *Apico1* | F: AAAATAACGCGAAAAGATTCA  R: TGTGGAAGATATGGAAATAAAGGA | F: CGTGCAAAACAATCATCAGA  R: GCAGAAAACGCTGATTTACCTT | 522 |
| *Apico2* | F: TCCTTTATTTCCATATCTTCCACA  R: AAAACCTTTAGTATGAAACGGTGAA | F: TTCCACATAATTTATCTCCAACTG  R: TGGATATGATTTTGAAGATGCTG | 489 |

Abbreviations: *SAG1*, surface antigen 1; *SAG2*, surface antigen 2; *SAG3*, surface antigen 3; *BTUB*, beta-tubulin; *GRA6*, dense granule antigen 6; *PK1*, protein kinase 1; *HP*, hypoxanthine-xanthine-guanine phosphoribosyltransferase; *EF*, elongation factor.

**Table S3.** Summary of genotyping of four *Toxoplasma gondii* isolates re-classified.

| Isolate ID | Host | Location | *SAG1* | 5'+3' *SAG2* | Alternative *SAG2* | *SAG3* | *BTUB* | *GRA6* | C22-8 | C29-2 | L358 | *PK1* | *Apico* | Genotype |
| --- | --- | --- | --- | --- | --- | --- | --- | --- | --- | --- | --- | --- | --- | --- |
| GT1 | Goat | United States | I | I | I | I | I | I | I | I | I | I | I | Reference, Type I, ToxoDB#10 |
| PTG | Sheep | United States | II/III | II | II | II | II | II | II | II | II | II | II | Reference, Type II, ToxoDB#1 |
| CTG | Cat | United States | II/III | III | III | III | III | III | III | III | III | III | III | Reference, Type III, ToxoDB#2 |
| MAS | Human | France | u-1^*^ | I | II | III | III | III | u-1^*^ | I | I | III | I | Reference, ToxoDB#17 |
| TgCgCa1 | Cougar | Canada | I | II | II | III | II | II | II | u-1^*^ | I | u-2^*^ | I | Reference, ToxoDB#66 |
| TgCatBr5 | Cat | Brazil | I | III | III | III | III | III | I | I | I | u-1^*^ | I | Reference, ToxoDB#19 |
| TgCatBr64 | Cat | Brazil | I | I | u-1 | III | III | III | u-1 | I | III | III | I | Reference, ToxoDB#111 |
| TgRsCr1 | Toucan | Costa Rica | u-1 | I | II | III | I | III | u-2 | I | I | III | I | Reference, ToxoDB#52 |
| TgC1 | Cat | Guangzhou, Guangdong | I | I | II | III | I | III | II | I | III | III | I | ToxoDB#29 |
| TgC2 | Cat | Guangzhou, Guangdong | I | II | II | III | II | II | II | I | III | II | I | ToxoDB#113 |
| TgCYn14 | Cat | Kunming, Yunnan | II | II | II | II | II | II | II | II | II | II | II | ToxoDB#1 |

^*^Note: u-1 and u-2 represent unique RFLP genotypes, respectively. Abbreviations: *SAG1*, surface antigen 1; *SAG2*, surface antigen 2; *SAG3*, surface antigen 3; *BTUB*, beta-tubulin; *GRA6*, dense granule antigen 6; *PK1*, protein kinase 1; RFLP, restriction fragment length polymorphism.

**References**

[1] Y.Y. Lu, H. Dong, Y.J. Feng, K. Wang, Y.B. Jiang, L.X. Zhang, et al., Avirulence and lysozyme secretion in Paneth cells after infection of BALB/c mice with oocysts of *Toxoplasma gondii* strains TgCatCHn2 (ToxoDB#17) and TgCatCHn4 (ToxoDB#9), Vet. Parasitol. 252 (2018) 1-8, doi: 10.1016/j.vetpar.2018.01.016.

[2] H. Ren, G. Mao, Y. Zhang, N. Zhu, Q. Liang, Y. Jiang, et al., Isolation and characterization of a viable *Toxoplasma gondii* from captive caracal (*Caracal caracal*), Pathogens 12 (12) (2023) 1412, doi: 10.3390/pathogens12121412.

[3] W. Qian, H. Wang, C. Su, D. Shan, X. Cui, N. Yang, et al., Isolation and characterization of *Toxoplasma gondii* strains from stray cats revealed a single genotype in Beijing, China, Vet. Parasitol. 187 (3-4) (2012) 408-413, doi: 10.1016/j.vetpar.2012.01.026.

[4] P. Zhou, H. Zhang, R.Q. Lin, D.L. Zhang, H.Q. Song, C. Su, et al., Genetic characterization of *Toxoplasma gondii* isolates from China. Parasitol. Int. 58 (2) (2009) 193-195, doi: 10.1016/j.parint.2009.01.006.

[5] J.P. Dubey, X.Q. Zhu, N. Sundar, H. Zhang, O.C. Kwok, C. Su. Genetic and biologic characterization of *Toxoplasma gondii* isolates of cats from China, Vet. Parasitol. 145 (3-4) (2007) 352-356, doi: 10.1016/j.vetpar.2006.12.016.

[6] Y.M. Tian, S.Y. Huang, Q. Miao, H.H. Jiang, J.F. Yang, C. Su, et al., Genetic characterization of *Toxoplasma gondii* from cats in Yunnan Province, Southwestern China, Parasit. Vectors 7 (2014) 178, doi: 10.1186/1756-3305-7-178.

[7] Z.W. Chen, J.M. Gao, X.X. Huo, L. Wang, L. Yu, F. Halm-Lai, et al., Genotyping of *Toxoplasma gondii* isolates from cats in different geographic regions of China, Vet. Parasitol. 183 (1-2) (2011) 166-170, doi: 10.1016/j.vetpar.2011.06.013.

[8] M.J. Chu, L.Y. Huang, W.Y. Miao, Y.F. Song, Y.S. Lin, S.A. Li, et al., First molecular detection and genotype identification of *Toxoplasma gondii* in chickens from Farmers’ Markets in Fujian Province, southeastern China, Pathogens 12 (10) (2023) 1243, doi: 10.3390/pathogens12101243.

[9] S.A. Li, L.Y. Huang, X.D. Guo, W.Y. Miao, Y.S. Lin, D.H. Zhou. First identified *Toxoplasma gondii* Type I in market-sold ducks in Fujian province, China: a significant for public health, Poult. Sci. 103 (9) (2024) 104024, doi: 10.1016/j.psj.2024.104024.

[10] E.K. Shwab, X.Q. Zhu, D. Majumdar, H.F. Pena, S.M. Gennari, J.P. Dubey, et al., Geographical patterns of *Toxoplasma gondii* genetic diversity revealed by multilocus PCR-RFLP genotyping, Parasitology 141 (4) (2014) 453-461, doi: 10.1017/S0031182013001844.

[11] R. Su, H. Dong, T. Li, Y. Jiang, Z. Yuan, C. Su, et al., *Toxoplasma gondii* in four captive kangaroos (*Macropus*spp*.*) in China: Isolation of a strain of a new genotype from an eastern grey kangaroo (*Macropus giganteus*), Int. J. Parasitol. Parasites Wildl. 8 (2019) 234-239, doi: 10.1016/j.ijppaw.2019.03.003.

[12] H.H. Jiang, S.Y. Qin, W. Wang, B. He, T.S. Hu, J.M. Wu, et al., Prevalence and genetic characterization of *Toxoplasma gondii* infection in bats in southern China, Vet. Parasitol. 203 (3-4) (2014) 318-321, doi: 10.1016/j.vetpar.2014.04.016.

[13] P. Zhou, H. Nie, L.X. Zhang, H.Y. Wang, C.C. Yin, C. Su, et al., Genetic characterization of *Toxoplasma gondii* isolates from pigs in China, J. Parasitol. 96 (5) (2010) 1027-1029, doi: 10.1645/GE-2465.1.

[14] H.H. Jiang, S.C. Wang, S.Y. Huang, L. Zhao, Z.D. Wang, X.Q. Zhu, et al., Genetic characterization of *Toxoplasma gondii* isolates from pigs in Jilin Province, Northeastern China, Foodborne Pathog. Dis. 13 (2) (2016) 88-92, doi: 10.1089/fpd.2015.2043.

[15] H.H. Jiang, S.Y. Huang, D.H. Zhou, X.X. Zhang, C. Su, S.Z. Deng, et al., Genetic characterization of *Toxoplasma gondii* from pigs from different localities in China by PCR-RFLP, Parasit. Vectors 6 (2013) 227, doi: 10.1186/1756-3305-6-227.

[16] Z.F. Hou, S.J. Su, D.D. Liu, L.L. Wang, C.L. Jia, Zhao ZX, et al., Prevalence, risk factors and genetic characterization of *Toxoplasma gondii* in sick pigs and stray cats in Jiangsu Province, eastern China, Infect. Genet. Evol. 60 (2018) 17-25. doi: 10.1016/j.meegid.2018.02.007.

[17] Y. Jiang, S. Xin, Y. Ma, H. Zhang, X. Yang, Y. Yang. Low Prevalence of *Toxoplasma gondii* in sheep and isolation of a viable strain from edible mutton from Central China, Pathogens 12 (6) (2023) 27, doi: 10.3390/pathogens12060827.

[18] Y. Yang, Y. Feng, Q. Yao, Y. Wang, Y. Lu, H. Liang, et al., Seroprevalence, isolation, genotyping, and pathogenicity of *Toxoplasma gondii* strains from sheep in China, Front. Microbiol. 8 (2017) 136, doi: 10.3389/fmicb.2017.00136.

[19] H. Ren, L. Yang, N. Zhu, J. Li, C. Su, Y. Jiang, et al., Additional evidence of tigers (*Panthera tigris altaica*) as intermediate hosts for *Toxoplasma gondii* through the isolation of viable strains, Int. J. Parasitol. Parasites. Wildl. 19 (2022) 330-335, doi: 10.1016/j.ijppaw.2022.11.009.

[20] Y. Yang, N. Jiang, S. Xin, L. Zhang. *Toxoplasma gondii* infection in white spoonbills (*Platalea leucorodia*) from Henan Province, China, Emerg. Microbes Infect. 9 (1) (2020) 2619-2621, doi: 10.1080/22221751.2020.1854057.

[21] L. Yang, H. Ren, N. Zhu, G. Mao, J. Li, C. Su, et al., Epidemiology and isolation of viable *Toxoplasma gondii* strain from macropods, Heliyon 9 (3) (2023) e13960, doi: 10.1016/j.heliyon.2023.e13960.
